# Investigating the impact of inter-limb asymmetry in hamstring strength on jump, sprint, and strength performance in young athletes: comparing the role of gross force

**DOI:** 10.3389/fphys.2023.1185397

**Published:** 2023-05-25

**Authors:** Dongting Jiang, Zijian Liu, Xiaoyu Ling, Jinjin Dai, Lijun Long, Yongren Lu, Shengqing Zhou

**Affiliations:** ^1^ Sports Coaching College, Beijing Sports University, Beijing, China; ^2^ Physical Education College, China University of Geosciences, Wuhan, China; ^3^ School of Sports Medicine and Rehabilitation, Beijing Sports University, Beijing, China; ^4^ College of Physical Education, Hunan Normal University, Changsha, China; ^5^ Youth Competitive Sports School, Guangdong Provincial Sports Bureau, Guangzhou, China; ^6^ School of Physical Education and Health, Guangxi Normal University, Guilin, China

**Keywords:** asymmetry, hamstring, kinetics, performance, youth athletes

## Abstract

The main purpose of this cross-sectional study was to examine the impact of the inter-limb asymmetry of hamstring strength on jump, sprint and strength performance and to compare the effects of inter-limb asymmetry of hamstring strength with gross force (GF) of the hamstring on these physical qualities in youth volleyball athletes. Eighty-one youth volleyball players (age: 16.6 ± 1.9 years; training experience: 3.0 ± 0.9 years; height: 191.4 ± 7.1 cm; body mass: 78.5 ± 12.9 kg; lean body mass: 63.5 ± 10.5 kg; body fat rate: 18.6% ± 6.1%) performed a mid-season battery of tests consisting of morphological test, depth jump (DJ), counter movement jump (CMJ), squat jump (SJ), 10 m sprint, isometric mid-thigh pull (IMTP) and hamstring strength test. All tests reported good to excellent reliability (ICC range = 0.815–0.996) and acceptable variability (CV range = 3.26–7.84%). Results show a significant negative relationship between inter-limb asymmetry of hamstring strength and all physical qualities (r = −0.271 to −0.445; *p* < 0.05), and a significant positive relationship between GF of hamstring and all physical qualities (r = 0.303 to 0.664; *p* < 0.05). Additionally, GF of hamstring was more relevant to IMTP-PF (peak force) (r = 0.664) and inter-limb asymmetry of hamstring strength was more relevant to 10 m sprint (r = −0.445). The findings from this study indicate that, for youth athletes, the GF of the hamstring is crucial for overall lower limb strength performance, and the importance of inter-limb symmetry of hamstring strength increases with the complexity of the task.

## Introduction

Strength asymmetry refers to a lack of equality between limbs or muscle groups and the impact of this asymmetry on injury risk and sports performance has been extensively investigated in the literature, particularly in strength and conditioning research (Keeley, Plummer, & Oliver, 2011; Parkinson et al., 2021). Evidence has confirmed that strength differences between limbs increase the risk of injury (Croisier et al., 2008; Brumitt et al., 2013). However, there is debate as to whether inter-limb strength asymmetry impairs sports performance. The literature suggests that inter-limb strength asymmetry appears to have a negative effect on performance in jumping, sprinting and change of direction (COD) (Bell et al., 2014; Bishop et al., 2018; Michailidis et al., 2020; Bishop et al., 2021). However, there is also evidence to suggest that inter-limb strength asymmetry may not always negatively impact performance (Lockie et al., 2014; Dos’Santos et al., 2018). For example, higher division soccer players have been found to show larger asymmetries compared to lower division players (Ferreira et al., 2018). Furthermore, inter-limb differences have been identified in a variety of sports including sprinting (Rumpf et al., 2014; Exell, Irwin, Gittoes, & Kerwin, 2017; Meyers, Oliver, Hughes, Lloyd, & Cronin, 2017), kickboxing (Stanton, Reaburn, & Delvecchio, 2015), swimming (Evershed, Burkett, & Mellifont, 2014), basketball (Schiltz et al., 2009), and rowing (Buckeridge, Hislop, Bull, & McGregor, 2012), suggesting that inter-limb asymmetry may be reasonable under certain circumstances. Therefore, further research is needed to explore the relationship between inter-limb strength asymmetry and sports performance.

Previous studies have highlighted the importance of hamstring function for athletic performance, especially in terms of explosive strength characteristics. Hamstrings are vital for sprint acceleration by increasing the production of horizontal ground reaction force (GRF) (Morin et al., 2015). Nordic hamstring exercise (NHE) training has been found to induce sustained improvements in hamstring function in athletes, which can be transferred to 5 m and 10 m sprint speeds as well as maximum countermovement jump (CMJ) height (Goran skok et al., 2017; Krommes et al., 2017; Ishøi et al., 2018; Timmins et al., 2021). Additionally, evidence suggests that hamstring strength is positively correlated with sprinting performance (Timmins et al., 2021). In addition to monitoring hamstring strength, recent research has also focused on examining inter-limb asymmetry of hamstring strength (Cuthbert et al., 2021; Bishop et al., 2022). Studies have indicated that inter-limb asymmetry of hamstring strength (>15%) may significantly increase an athlete’s risk of hamstring injury (Croisier et al., 2008; Kyritsis et al., 2016). However, it is unclear whether inter-limb asymmetry of hamstring strength has negative effects on sports performance, including jump, sprint, and strength performance, beyond increasing the risk of injury.

Given that inter-limb strength asymmetry may affect health and performance, and hamstring strength plays an important role in athletic performance, inter-limb asymmetry of hamstring strength has been proved to be harmful to the safety of hamstring muscle during exercise, further studies are needed to determine its impacts on athletic performance, which could help to further clarify the role of inter-limb asymmetry of hamstring strength and its impacts on performance. Therefore, the aims of the present study were 2-fold: 1) to examine the effects of the inter-limb asymmetry of hamstring strength on jump, sprint and strength performance and, 2) to compare the difference between the effects of inter-limb asymmetry of hamstring strength and gross force (GF) of hamstring on these physical qualities. It was hypothesized that the larger inter-limb asymmetry of hamstring strength would be correlated with reduced physical performance and inter-limb asymmetry of hamstring strength played a different role in these athletic performance than GF of hamstring. Given that inter-limb strength asymmetry has been shown to potentially impact health and athletic performance, and hamstring strength is known to play a crucial role in physical performance, it is important to investigate the effects of inter-limb asymmetry of hamstring strength on athletic performance. Specifically, understanding the relationship between inter-limb asymmetry of hamstring strength and jump, sprint, and strength performance may help to clarify the role of this asymmetry and its impact on athletic performance. Therefore, the present study aims to achieve two goals: 1) to examine the impact of inter-limb asymmetry of hamstring strength on jump, sprint, and strength performance and 2) to compare the effects of inter-limb asymmetry of hamstring strength with gross force (GF) of the hamstring on these physical qualities. Our hypothesis was that greater inter-limb asymmetry of hamstring strength would be associated with decreased physical performance, and that inter-limb asymmetry of hamstring strength played a different role in athletic performance compared to GF of the hamstring.

## Methods

### Participants

Eighty-one youth volleyball players (age: 16.6 ± 1.9 years; training experience: 3.0 ± 0.9 years; height: 191.4 ± 7.2 cm; body mass: 78.5 ± 12.9 kg; lean body mass: 63.5 ± 10.5 kg; body fat rate: 18.6% ± 6.1%) volunteered for this study. Of the participants, 60 were male (age: 16.6 ± 1.9 years; training experience: 3.0 ± 0.9 years; height: 194.1 ± 5.8 cm; body mass: 80.6 ± 12.8 kg; lean body mass: 66.8 ± 9.4 kg; body fat rate: 15.9% ± 4.2%) and 21 were female (age: 16.4 ± 2.1 years; training experience: 3.0 ± 0.9 years height: 183.5 ± 4.5 cm; body mass: 72.4 ± 11.3 kg; lean body mass: 54.0 ± 7.5 kg; body fat rate: 26.3% ± 3.5%).

The local volleyball club policy required players to perform 3–4 volleyball drills per week and at least 2 structured strength and conditioning sessions per week during the testing cycle. All subjects met the following inclusion criteria: a) a background of ≥2 years of systematic volleyball training and competitive volleyball match experience, b) continuous volleyball training for the previous 2 months without having sustained any musculoskeletal injuries, c) the absence of potential medical problems that could compromise participation or performance in the study, and d) the absence of any lower-extremity injury in the past 3 months.

All subjects provided written informed consent and their personal information was handled anonymously, and parental or guardian consent for all subjects under the age of 18 involved in this investigation were obtained. The study received ethical approval from the Sports Science Ethics Committee of Beijing Sports University. To calculate the sample size, a free statistical software (G * Power, v.3.1.9.7, Dusseldorf, Germany) was used. Given the applied a bivariate linear regression, a medium overall effect size (ES) = 0.4, an alpha-error = 0.05 and the desired power (1-ß error) = 0.8, the total sample size resulted in 44 participants (Beck, 2013). To reduce the risk of experimental mortality we recruited a larger sample.

### Experimental design

This study employed a cross-sectional design and recruited youth volleyball players from several local volleyball clubs. This experiment was conducted from April to May 2022, with a total duration of 4 weeks, consisted of multiple assessments including morphological test, depth jump (DJ), counter movement jump (CMJ), squat jump (SJ), 10 m sprint, isometric mid-thigh pull (IMTP) and hamstring strength test.

Prior to the test, all participants were familiarized with the entire testing programs, and their information was recorded according to the study requirements. To ensure the validity and reliability of the test data, all subjects underwent a standardized warm-up protocol. Moreover, participants were instructed to avoid any strenuous physical activity 48 h before testing and maintain their usual diet.

### Procedures

Due to the large number of participants and testing items in this experiment, the testing period lasted for 4 weeks. All participants performed testing items according to a standardized warm-up procedure and testing sequence. A rest interval of at least 2 min was allowed between each physical fitness trial. During the waiting time, participants engaged in low-intensity activities, such as walking and jogging, to keep themselves prepared for the subsequent test. A warm-up consisting of 10 min of submaximal running and specific exercises (i.e., submaximal vertical and horizontal jumps) was carried out before each testing session. The testing sequence was as follows: morphological test, DJ, CMJ, SJ, 10 m sprint, IMTP and hamstring strength test.

#### Morphological test

Stature was measured using a stadiometer (Bodymeter 206; SECA, Hamburg, Germany), while lean, fat, and total mass distribution were assessed utilizing DEXA (DXA; Hologic Discovery A, Waltham, MA, United States). Participants were instructed to wear minimal garments made of light fabric, free of metal components (zippers, wires, or fasteners), and to remove metallic items from their pockets before lying in a supine position on the scanning bed with both arms pronated to their side. To guarantee consistent and reproducible positioning, participants were aided in aligning their head in a straight line with their torso and pelvis, internally rotating and securing their legs and feet at a 45° angle, and placing their arms adjacent to their body within the scanning area (Stewart and Hannan, 2000). Subsequently, full-body scan images were analyzed using proprietary software (version 12.4; QDR for Windows, Hologic, Waltham, MA, United States).

#### Depth jump (DJ), countermovement jump (CMJ) and squat jump (SJ)

Jump tests were conducted using the ForceDecks FD4000 dual force platforms (Vald Performance, Brisbane, Queensland, Australia) sampling at 1,000 Hz, which has been previously proposed as a reliable device (CV < 10%, ICC >0.70; Heishman et al., 2020). The commercially available ForceDecks software (Vald Performance, Brisbane, Queensland, Australia) was used to analyse and generate the jumping variables using conventional methods. To perform the SJ, athletes stood in a comfortable bilateral position in the center of the ergometer with hands at their waist and then assumed a half-squat position for 2–3 s. When instructed, athletes accelerated vertically upward from the half-squat position, jumped as fast as possible, and returned to an upright standing position. Similar procedures were followed for the CMJ and DJ. For the CMJ, athletes stood in a comfortable bilateral position in the center of the ergometer with their hands secured at the waist. When instructed, athletes bent at the hips and knees to a depth of their choice and then accelerated vertically to jump as fast as possible. For the DJ, athletes stood on a 45 cm jumping box with one foot hanging in the air and their hands fixed at their waist. When instructed, athletes naturally jumped down, landed on both feet, and then jumped up as fast as possible, finishing the jump with both feet landing at the same time. Each jump test was repeated three times with 1–2 min of rest between each trial. Athletes returned to the starting position and held it for 2–3 s to prepare for the next jump. The height of the jumps was recorded in centimeters (cm) for all trials, and the highest jump was subsequently used for further analysis. Athletes were instructed to jump as high as possible and to extend their legs as fast as possible to maximize explosive power. If an athlete’s hand left their waist, the jump was considered invalid and restarted after 1–2 min of rest.

#### 10 m sprint

Linear speed was measured using a 10 m sprint test with a SMARTSPEED timing gate system (Fusion Sport, Queensland, Australia), which included an electric light gate placed at the starting and ending points. The timing system selected has been proven to be reliable in previous studies (CV = 0.6–4.2%, ICC = 0.82–0.97; Austin et al., 2013). The sprint test was conducted on the outdoor athletic track. The electro-optical gate sensors were connected to the SMARTSPEED timing gate system software to collect data. Athletes started in a standing position 0.5 m behind the first photoelectric gate and upon instruction, sprinted as quickly as possible for 10 m. The timing was accurate to one 100th of a second. Athletes performed three trials with 60-s rest intervals between each trial, and the fastest trial was selected for further analysis.

#### Isometric mid-thigh pull (IMTP)

The IMTP test was conducted using dual force plates (Force Decks, VALD Performance, FD4000, Queensland, Australia) sampling at 1,000 Hz, which has demonstrated sufficient reliability (CV = 5%, ICC = 0.94; Ritti-Dias et al., 2011). To ensure consistent positioning, the mid-thigh position was marked for each participant as the midpoint between the knee and hip joints. The barbell height was then adjusted to align with the mid-thigh. Participants stood on the force platform and grasped the barbell with an overhand grip, ensuring that the knee joint was within 120°–145° and the hip joint was within 140°–150° using a protractor. They were strapped to the barbell in a manner similar to previous investigations (Dos' Santos et al., 2017; Haff et al., 2005), with the torso upright and the knee and hip angles maintained during testing. At the start signal, athletes exerted maximal force as quickly as possible and sustained the force for 6 s without leaning forward or backward. All athletes were instructed to relax before the command “GO!” to avoid precontraction. Peak force was calculated as the highest force achieved during the 6-s isometric test, adjusted for body weight in Newtons. Each athlete performed three trials of the IMTP test, with 2–3 min of rest between trials. The highest peak force achieved across the three trials was used for further analysis.

#### Hamstring strength test

The NordBord device (Vald Performance, Newstead, Australia) was used to assess hamstring strength with a sampling frequency of 50 Hz. The reliability of this assessment has been described (ICC = 0.83–0.90, typical error = 21.7–27.5; van Dyk et al., 2018). Prior to the test, athletes completed a 5-min warm-up on a stationary bike with an RPE between 5 and 10, followed by 1 × 10 repetitions of dynamic stretches, including multiplanar lunges, multiplanar leg swings, body mass squats, and the “world’s greatest stretch”. During the test, each athlete was positioned in a kneeling position on the NordBord device’s pads, with their ankles secured by a single hook perpendicular to the ground, which was attached to a custom single-axis load cell with wireless data acquisition capability. The athlete raised their head and chest, maintained an upright body posture with straight trunk and hips, and extended their knees to lean forward. The hamstrings fired to resist a rapid descent, and the athlete’s palms faced forward while bending their elbows until their palms touched the ground. Athletes completed a warm-up set of three attempts without data collection, followed by a 3-min rest period. Then, a formal single set of three hamstring strength tests was performed for data collection. Test scores were output as the maximum force in Newtons (N) for each limb, and the GF of hamstring was calculated as the sum of the maximum force of each limb. The mean of the three trials was used for further analysis.

### Statistical analyses

All data was recorded as mean and SD. Data were analyzed by the IBM SPSS Statistics (version 24.0; SPSS, Inc., Armonk, NY, United States). Normality of the data was confirmed (*p* < 0.05) using the Shapiro-Wilk test. Absolute and relative reliability was calculated via the coefficient of variation (CV) and intraclass correlation coefficient (ICC) with absolute agreement (95% confidence intervals), respectively. CV values <10% were considered acceptable (Cormack et al., 2008) and ICCs were interpreted in line with previous suggestions from Koo and Li (2016) where values >0.9 = excellent, 0.75 to 0.9 = good, 0.5 to 0.74 = moderate, and <0.5 = poor. Independent-samples t-tests were used to determine possible differences in all fitness test between males and females. Practical differences were assessed using Hedges’ g effect size (ES) data, and were interpreted in line with suggestions by Hopkins et al. (2009) where 0.00 to 0.19 = trivial, 0.20 to 0.59 = small, 0.60 to 1.19 = moderate, 1.20 to 1.99 = large, and ≥2.00 = very large. Pearson’s r correlations were conducted to determine the relationships between each sports performance test with inter-limb asymmetry of hamstring strength and GF of hamstring, with statistical significance set at *p* < 0.05. Magnitudes of correlation were interpreted as 0.00 to 0.09 = trivial, 0.10 to 0.29 = small, 0.30 to 0.49 = moderate, 0.50 to 0.69 = large, 0.70 to 0.89 = very large, 0.90 to 0.99 = nearly perfect, and 1.00 = perfect (Hopkins et al., 2009). Interlimb asymmetries were quantified using a standard percentage difference equation: 100/(max value) * (min value)*-1 + 100 (Bishop et al., 2021; Cuthbert et al., 2021).

## Results

81 participants completed all fitness tests, including 60 male athletes (age: 16.6 ± 1.9 years; training experience: 3.0 ± 0.9 years; height: 194.1 ± 5.8 cm; body mass: 80.6 ± 12.8 kg; lean body mass: 66.8 ± 9.4 kg; body fat rate: 15.9% ± 4.2%) and 21 female athletes (age: 16.4 ± 2.1 years; training experience: 3.0 ± 0.9 years height: 183.5 ± 4.5 cm; body mass: 72.4 ± 11.3 kg; lean body mass: 54.0 ± 7.5 kg; body fat rate: 26.3% ± 3.5%) (Table 1).

**TABLE 1:** Baseline data of the subjects.

Basic information	Total (n = 81)	Male (n = 60)	Female (n = 21)
Age (years)	16.6 ± 1.9	16.6 ± 1.9	16.4 ± 2.1
Training experience (years)	3.0 ± 0.9	3.0 ± 0.9	3.0 ± 0.9
Height (cm)	191.4 ± 7.2	194.1 ± 5.8	183.5 ± 4.5
Body mass (kg)	78.5 ± 12.9	80.6 ± 12.8	72.4 ± 11.3
Lean body mass (kg)	63.5 ± 10.5	66.8 ± 9.4	54.0 ± 7.5
Body fat rate (%)	18.6 ± 6.1	15.9 ± 4.2	26.3 ± 3.5

Mean test scores and ES between male and female groups are presented in Table 2. The results between male and female groups for all test were significant (*p* < 0.05). The ES of Hamstring-Asymmetry and IMTP-PF between male and female groups showed moderate standard with 0.55 and 0.78, respectively. The ES of all other indicators showed large standard.

**TABLE 2:** Mean testing data ± standard deviations for each group and effect sizes between male and female groups.

Fitness test	Total (n = 81)	Male (n = 60)	Female (n = 21)	Male vs. Female (ES)
Hamstring-Left (N)	366.25 ± 91.95	388.12 ± 88.08	303.81 ± 73.54	1.00 (large)***
Hamstring-Right (N)	358.28 ± 96.12	380.65 ± 93.85	294.38 ± 72.01	0.97 (large)***
Hamstring-GF (N)	724.54 ± 185.19	768.77 ± 179.06	598.19 ± 141.30	1.00 (large)***
Hamstring-Asymmetry (%)	7.50 ± 5.90	6.67 ± 5.68	9.87 ± 6.02	0.55 (moderate)*
DJ (cm)	33.94 ± 6.91	36.05 ± 6.28	27.90 ± 4.79	1.37 (large)***
CMJ (cm)	34.44 ± 7.12	36.76 ± 6.42	27.84 ± 4.38	1.49 (large)***
SJ (cm)	31.26 ± 6.82	33.29 ± 6.32	25.45 ± 4.53	1.32 (large)***
10 m-Sprint (s)	1.98 ± 0.15	1.91 ± 0.10	2.17 ± 0.11	2.53 (large)***
IMTP-PF (N)	2814.94 ± 856.30	2980.17 ± 856.65	2342.86 ± 672.48	0.78 (moderate)**

GF, gross force; DJ, depth jump; CMJ, counter movement jump; SJ, squt jump; IMTP, isometric mid-thigh pull; PF, peak force; ES, effect size.

*p<0.05; **: *p* < 0.01; ***: *p* < 0.001.

Table 3 shows mean test scores and SD for each test and accompanying reliability data using the CV and ICC. For absolute reliability, all the performed tests had acceptable between-trial consistency with all CV values below 8%. For relative reliability, all ICC values were found to be excellent (ICC range = 0.918–0.978), with the exception of the 10 m sprint, which showed a slightly lower ICC of 0.815.

**TABLE 3:** Mean test scores and standard deviations, inter-limb asymmetry, and accompanying reliability data for each test.

Fitness test	Mean ± SD	Asymmetry (%)	CV(%)	ICC(95% CI)
Hamstring-Left (N)	366.25 ± 91.95	7.50 ± 5.90	6.94	0.925 (0.890–0.949)
Hamstring-Right (N)	358.28 ± 96.12		7.83	0.918 (0.884–0.944)
Hamstring-GF (N)	724.54 ± 185.19	—	5.24	0.958 (0.935–0.973)
DJ (cm)	33.94 ± 6.91	—	3.71	0.969 (0.809–0.989)
CMJ (cm)	34.44 ± 7.12	—	3.24	0.978 (0.882–0.992)
SJ (cm)	31.26 ± 6.82	—	3.63	0.976 (0.839–0.991)
10 m-Sprint (s)	1.98 ± 0.15	—	3.26	0.815 (0.341–0.927)
IMTP-PF N)	2814.94 ± 856.30	—	5.39	0.976 (0.813–0.992)

GF, gross force; DJ, depth jump; CMJ, counter movement jump; SJ, squt jump; IMTP, isometric mid-thigh pull; PF, peak force; SD, standard deviation; CV, coefficient of variation; ICC, intraclass correlation coefficien; CI, confidence interval.

Table 4 shows the Pearson’s r correlations between inter-limb asymmetry scores and GF of hamstring with each test data. Significant negative correlations were shown for total group between asymmetry and all physical qualities (*p* < 0.05), and significant positive correlations were shown for total group between GF and all physical qualities (*p* < 0.05). In the male group, asymmetry showed a significant negative correlation with SJ (r = −0.361; *p* = 0.005) and 10 m sprint (r = −0.471; *p* < 0.001) performance, respectively, and GF showed a significant positive correlation with the IMTP-PF (r = 0.648; *p* < 0.001) performance. In the female group, asymmetry showed a significant negative correlation with DJ (r = −0.491; *p* = 0.024) and IMTP-PF (r = −0.460, *p* = 0.036) performance, respectively, and GF showed a significant positive correlation with IMTP-PF (r = 0.458; *p* = 0.037) performance.

**TABLE 4:** Correlation between inter-limb asymmetry of hamstring strength and gross force of hamstring with performance scores on each test.

Fitness test	Hamstring-asymmetry			Hamstring-GF		
	Total (n = 81)	Male (n = 60)	Female (n = 21)	Total (n = 81)	Male (n = 60)	Female (n = 21)
DJ (cm)	−0.308**	−0.150	−0.491*	0.339**	0.161	0.178
CMJ (cm)	−0.329**	−0.211	−0.375	0.340**	0.180	0.043
SJ (cm)	−0.394***	−0.361**	−0.202	0.303**	0.140	0.081
10 m-Sprint (s)	−0.445***	−0.471***	−0.247	0.328**	0.032	0.321
IMTP-PF N)	−0.271*	−0.141	−0.460*	0.664***	0.648***	0.458*

GF, gross force; SJ, squat jump; CMJ, counter movement jump; DJ, depth jump; IMTP, isometric mid-thigh pull; PF, peak force.

*p<0.05; **: *p* < 0.01; ***: *p* < 0.001.

## Discussion

The aim of this study was to investigate the effects of inter-limb asymmetry of hamstring strength and GF of hamstring on jump, sprint, and strength performance, and to compare the differences between them. The results indicated that inter-limb asymmetry of hamstring strength had a greater impact on sprint performance, while GF of hamstring was found to have a greater influence on strength performance. Interestingly, inter-limb asymmetry was just as important as GF for jump performance.

Mean data for all test protocols, including test reliability and mean inter-limb asymmetry values, were calculated and reported in Table 3. All tests showed good to excellent reliability (ICC range = 0.815–0.996) and acceptable variability (CV range = 3.26–7.84%), indicating that results can be interpreted with confidence. Furthermore, this study examined gender differences in each test, and the results showed statistically significant differences between male and female athletes. Regarding the correlations between each indicator with inter-limb asymmetry of hamstring strength and GF of hamstring, the results were found to be inconsistent between males and females. Due to the large difference in sample size between the genders, the analysis of different characteristics between them was restricted, which is also a limitation of this study. Moreover, the primary aim of this study was to determine the effects of inter-limb asymmetry of hamstring strength and GF of hamstring on jump, sprint, and strength performance, rather than comparing the differences between genders. Therefore, further investigation is needed to examine gender differences. It is also important to note that long-term specialized training may induce the adaptation of inter-limb asymmetry (Maloney, 2019), which could limit the true reflection of the relationship between inter-limb asymmetry and physical performance. As a result, this study chose youth athletes with relatively less training experience as experimental subjects.

The present study found that inter-limb asymmetry of hamstring strength had a greater impact on sprint performance than GF. It is worth noting that the tests in this experiment were performed using bilateral movements, except for the 10 m sprint. Sprinting is a typical alternating limb movement and requires high levels of stability and neuromuscular control (Struzik et al., 2016). Gonzalo-Skok et al. (2017) reported that unilateral resistance training could effectively reduce inter-limb asymmetry and lead to greater enhancements in actions that require unilateral force application. Therefore, reducing inter-limb asymmetry may be beneficial for improving unilateral movements. Although Meyers et al. (2017) reported weak relationships (r = −0.24 to 0.39; *p* < 0.05) between various asymmetry metrics (step frequency, step length, flight time, and vertical stiffness) and sprint velocity in youth athletes (344 school aged boys; 11–16 years), large kinetic asymmetries did not appear to be detrimental to mean sprint velocity in sprint-trained athletes (Bishop et al., 2018). However, as the participants in this study were youth athletes (16.56 ± 1.93 years) without professional sprint training, inter-limb asymmetry of hamstring strength may impair sprint performance. During sprinting, the average activity in hamstring muscles was 33% greater in comparison to the quadriceps muscles, which meant that the greater involvement of hamstring muscles in this motor task (Pietraszewski et al., 2020). Accordingly, we suggested that reducing the inter-limb asymmetry of hamstring strength may effectively enhance sprint performance by improving the efficiency of alternating limb movements, especially for the youth cohort.

Compared to the small correlation observed between inter-limb asymmetry of hamstring strength and IMTP-PF (r = −0.271; *p* < 0.05), GF of the hamstring showed a large correlation with IMTP-PF (r = 0.664; *p* < 0.001). Anderson and Behm (2004) reported that unstable isometric maximum force output was 59.6% lower than under stable conditions. Since IMTP can evaluate the maximum strength of the lower limbs in an extremely stable condition, this may partly offset the negative impact of inter-limb asymmetry of hamstring strength. Thus, when performing tasks on a stable plane, GF of hamstring muscles appeared to be a more critical factor in determining strength performance than inter-limb asymmetry of hamstring strength. However, in jump tests, inter-limb symmetry of hamstring strength and GF of hamstring appeared to be equally important to jump performance. From a kinetic chain perspective, both sprint and jump tests belong to a closed kinetic chain (CKC) action, whereas the IMTP test is an open kinetic chain (OKC) action. Compared to OKC, CKC requires more intermuscular coordination and neuromuscular control (Karandikar and Vargas, 2011). In all physical qualities, only sprint is both a unilateral and OKC exercise, making it more relevant to inter-limb symmetry of hamstring strength than jump and strength performance. Therefore, we deduced that the importance of inter-limb symmetry of hamstring strength appeared to increase with the complexity of the task. Bishop et al. (2018) and Madruga-Parera et al. (2020) also indicated that strength asymmetries appeared to have a negative effect on performance tasks, including jumping, change-of-direction speed (CODS) and repeated sprint performance, thus minimising these differences would be beneficial.

In practical applications concerning sports injury prevention and performance improvement, hamstring GF seems to attract more attention than inter-limb asymmetry in hamstring strength. Numerous studies had validated that the increasement of hamstring strength had a positive impact on sprint and jump performance (Markovic et al., 2020; Váczi et al., 2022). However, studies examining inter-limb asymmetry of hamstring strength are still in the early stages, and the majority of them have focused on the prevention of sports injuries. It is commonly held that large inter-limb asymmetry may increase the risk of injury (Bishop et al., 2018), and sufficient hamstring strength could effectively prevent injuries during running or sprinting (Bishop et al., 2022). Therefore, from a health perspective, it is necessary to boost the hamstring strength and properly reduce inter-limb asymmetry of hamstring strength for running and sprinting. From a sports performance perspective, we also believe that increasing inter-limb symmetry of hamstring strength is critical. Based on the findings of this study and previous research, it is reasonable to speculate that more complex tasks may require greater inter-limb symmetry of hamstring muscles. A more symmetrical hamstring strength may contribute to improved overall stability in movements, help maintain proper technique, and enhance athletic performance. As a consequence, bilateral hamstring strength and inter-limb asymmetry of hamstring strength can affect movement efficiency in different ways. When bilateral hamstring strength approaches the limits of human body, appropriately reducing inter-limb asymmetry of hamstring strength may be the key to achieving a breakthrough, particularly for youth athletes.

Despite the usefulness of these findings, this study presents some limitations. Firstly, only a preliminary correlation analysis and a data-based theoretical inference were performed, therefore, more specific evidence is needed to verify whether reducing inter-limb asymmetry of hamstring strength can enhance the adaptability of complex tasks for youth athletes. Additionally, the research pointed out that asymmetries are an adaptive consequence that is magnified with long-standing sporting participation (Maloney, 2019), and may have no or active effects on the sports performance (Bishop et al., 2018). This finding may conflict with the results obtained for the youth cohort, so it is necessary to compare the difference between athletes of different levels. Moreover, there are many ways to calculate asymmetry, and other formulas could have let to different findings. Indeed, as absolute symmetry is almost non-existent, further exploration is required to determine what range of inter-limb asymmetry of hamstring strength is safe and beneficial for sports performance.

In summary, the results of this study indicate that the GF of the hamstring is crucial for overall lower limb strength performance, and the importance of inter-limb symmetry of hamstring strength increases with the complexity of the task. Therefore, we suggest that for youth athletes, it is important to concurrently develop hamstring strength and reduce inter-limb asymmetry of hamstring strength to enhance sprint and jump performance.

## Data Availability

The original contributions presented in the study are included in the article/supplementary material, further inquiries can be directed to the corresponding authors.
